# Navigating Pregnancy and the Healthcare System during COVID-19: A Qualitative Study with Perinatal Women of Color

**DOI:** 10.3390/ijerph192013698

**Published:** 2022-10-21

**Authors:** Tuyet-Mai H. Hoang, Wan-Jung Hsieh, B. Andi Lee, Kaylee Marie Lukacena, Karen M. Tabb

**Affiliations:** 1 School of Social Work, University of Illinois Urbana-Champaign, Urbana, IL 61801, USA; 2 Department of Social Work, National Taiwan University, Taipei 10617, Taiwan; 3 Department of Psychology, University of Illinois Urbana-Champaign, Champaign, IL 61820, USA; 4 Center for Social and Behavioral Science, University of Illinois Urbana-Champaign, Urbana, IL 61801, USA

**Keywords:** COVID-19, healthcare services, perinatal, Women of Color, intersectionality

## Abstract

Objective: To address health disparities in the perinatal period (i.e., during pregnancy and through one year after birth) by exploring the intersectional experiences of perinatal Black, Indigenous, and other People of Color (BIPOC) women during the COVID-19 pandemic. In this study, participants were asked if and how COVID-19 had impacted their experiences of receiving healthcare, whether they had faced any challenges during this time, how they had navigated these challenges, and what recommendations they had for improving perinatal healthcare. Methods: Between November 2021 and March 2022 our team conducted eight virtual focus groups comprising perinatal BIPOC women. A semi-structured interview protocol was used, and interviews were voice recorded and transcribed verbatim. The data were analyzed using reflexive thematic analysis. Results: Three major themes common in BIPOC perinatal healthcare experiences during COVID-19 were generated through engaging in reflexive thematic analysis: (1) an overwhelming lack of support from providers, (2) experiences of blame and shame, and (3) difficulties navigating institutional policies that were unclear or ever-changing during the COVID-19 pandemic. Recommendations from participants included greater empathic communication from providers in the face of uncertainty during COVID-19, greater access to information and guidance for caring for themselves and their babies, and an overall request for greater compassion while navigating an exciting and busy time. Relevance: These findings have implications for trauma-informed and inclusive perinatal care that can reduce the impacts of systemic inequalities for perinatal BIPOC women. This study offers a discussion of implications for future training for maternal health providers and implications for community-based programs.

## 1. Introduction

The Coronavirus disease 2019 (COVID-19) has magnified maternal health disparities to an unprecedented level, where pregnant people have difficulty accessing quality maternal care, resources, and support [1]. Non-consistent responses during COVID-19 from Washington, D.C., and the CDC yielded increased uncertainty and a lack of clear guidelines on response protocols and treatments for regional and local healthcare institutions. This resulted in barriers and delays in accessing services and resources for vulnerable populations. Conflicting healthcare information and misinformation amidst the changes in United States political leadership also put vulnerable populations, such as perinatal women, at the highest risk [2]. Recent data show an alarming increase of 33% in maternal deaths during the COVID-19 pandemic [3]. Particularly among racially minoritized women, non-Hispanic Black, American Indian/Alaska Native, and Asian/Pacific Islander women face a higher risk of death due to pregnancy-related factors compared to non-Hispanic White women [4,5]. The outlook is dimmer in the global context, where the average maternal mortality ratio has decreased in the past 25 years by 45%, but maternal mortality in the U.S. has increased by 58% [6]. In the U.S., systemic racism coupled with multiple forms of oppression put perinatal Black, Indigenous, and other People of Color (BIPOC) women in a uniquely vulnerable position during the pandemic [7], especially when they have higher lifetime exposures to chronic stressors [8,9]. Although emerging research has begun to shine a light on the impact of different systems of oppression on maternal health disparities [10,11,12], the direct causes of these disparities remain unclear across different minoritized groups. This is because perinatal BIPOC women are exposed to more pregnancy-related types of oppression and trauma compared to their counterparts [13,14,15], and the intersection of these social positionings (i.e., race, ethnicity, socioeconomic status, pregnancy, etc.) can produce a unique and challenging experience for each group of BIPOC women. This study explored the perinatal experiences of BIPOC women in seeking and accessing healthcare services during the pandemic. We understand that there is variation in the definition of BIPOC women, and in our study, we define BIPOC as self-identified racially minoritized women including Black, Asian, Pacific Islander, Hispanic/Latinx/Latina, Indigenous, multi-racial, and mixed race.

### Applying Intersectionality and Trauma-Informed Framework to Understand Perinatal Disparities

Intersectionality theory [16] was developed as a framework for understanding the intersecting impacts of both racism and sexism experienced by Black women. The current study utilized intersectionality theory as the lens for how we interpreted and analyzed our data from BIPOC perinatal people. Particularly, we made sense of the data through our considerations of power structures, systemic issues in healthcare settings, and historical and contemporary contexts of different forms of inequality based on participants’ social positionings. In addition, we grounded our interpretation within a trauma-informed framework to examine the disruptions in perinatal care during the COVID-19 pandemic and BIPOC women’s childbirth experiences. This study adopted a trauma-informed approach, with an emphasis on recognizing and addressing symptoms of trauma while providing care, to understand BIPOC women’s prior birth trauma and prior life adversities that contributed to their current traumatic stress [17].

Many existing studies examined the cumulative impact of social factors and determinants on perinatal health outcomes, yet this line of research has lost meaning within systems of care, especially when uncovering systemic factors that underlie the distribution of disparities [18]. More recently, scholars have called for a deeper approach to understanding the structural forces that shape the experiences of women’s health disparities [18], and a few recent studies have examined the impact of different forms of structural inequality in perinatal mortality, mental, and physical health outcomes [15]. The majority of the research studies that have used the intersectional framework have focused on non-Hispanic Black and non-White Hispanic populations, given the high disparities in these communities [19,20,21,22,23]. Fewer studies have included American Indian/Alaska Native, Asian/Pacific Islander, multiracial, or even nonbinary populations with underlying trauma history [12,15,24,25].

In addition, many quantitative studies have relied on demographic indicators to examine the health risks without examining structural factors, interactions of systemic forces, or power dynamic issues within the healthcare setting. Studies have also often focused on specific periods of the pregnancy or postpartum periods, or specific birth outcomes, and have not looked at the overall healthcare experiences of perinatal people, including their health and mental health outcomes. As the U.S healthcare system continues to prepare for new public health crises, existing challenges related to health disparities are exacerbated while new issues related to the pandemic response remain [1,26]. There is an urgent need to learn about the intersectional experiences of perinatal BIPOC women to address disparities and reduce preventable morbidity and mortality outcomes.

Our research questions were: (1) How did COVID-19 impact the experiences of accessing and receiving care for perinatal BIPOC women? (2) What were the unique challenges and systemic issues that BIPOC women encountered in seeking maternal care? (3) How did perinatal BIPOC women navigate these challenges within the U.S. healthcare system? and (4) What recommendations did perinatal BIPOC women have for improving current care? The goal of participant discussions was thus to explore the unique experiences and challenges perinatal BIPOC women encountered during the COVID-19 pandemic and solicit their recommendations for improving the healthcare system and addressing health disparities. The current study identified unique challenges that perinatal BIPOC women faced and highlighted their recommendations to improve care during the COVID-19 pandemic and in the U.S. healthcare system overall.

## 2. Materials and Methods

### 2.1. Data Collection and Procedure

This study used a purposeful sampling method to identify perinatal BIPOC women through our collaborator, the Illinois All Our Kids Early Childhood Networks initiative (AOK Networks) and public health listservs across the United States. Recruitment materials were distributed through emails, word of mouth, community partners, and referrals through AOK Networks and public health departments. Furthermore, prior to data collection, we engaged with a local perinatal network to refine our interview questions and research implementation. Inclusion criteria included people who (1) were at least 18 years of age, (2) self-identified as BIPOC women, and (3) were either in the second or third trimester of pregnancy or had delivered a child within the past 12 months. Exclusion criteria included people who (1) did not understand English and (2) were more than 12 months postpartum. The research team first scheduled a phone screening interview with each participant to determine their eligibility based on our inclusion and exclusion criteria. After participants agreed to join the focus group, a secure online consent form and a short demographic survey were sent to their provided email addresses. All participants completed the consent form before the focus groups took place. At the beginning of each focus group, consent was reviewed once more, and participants were reminded that they could discontinue participation at any point if they were not comfortable for any reason and provided verbal consent again for audio recording. Participants received a $50 gift card in appreciation for their time. All focus groups were conducted via HIPAA-secured Zoom calls and audio recording was used. The first and last authors moderated each of the eight focus groups. We received approval from our institutional review board prior to data collection.

### 2.2. Sample

We conducted eight focus groups with 41 participants from across the United States. The demographic characteristics of the participants are shown in Table 1. Participants were between the ages of 19 and 45 years, with the majority of participants in the 25- to 34-year range (*n* = 24; 59%). With regard to race/ethnicity, about half of the participants self-identified as African American/Black (*n* = 20; 49%), almost a fourth of the participants identified as Latina (*n* = 10; 24%), 20% identified as Asian/Asian Pacific Islander (*n* = 8), two identified as multiracial, and one participant identified as Indigenous. Geographically, most of the participants were living in the Midwest (*n* = 24; 58%) at the time of the study.

### 2.3. Data Analysis

We grounded our study in intersectionality and trauma-informed frameworks, which employ both feminist and critical epistemologies as well as an underlying assumption of existing experiences of trauma to explore and interpret the data. For this reason, we chose to conduct a reflexive thematic analysis, which provided flexibility for the intersectionality framework and valued the researcher’s positionalities and subjectivity as sources of knowledge production [27]. This analytic method allowed the greatest flexibility in reflecting on the researchers’ role in the research process and their influence on interpersonal interactions with participants. Our data analytic process followed six steps: (1) data familiarization, (2) initial code generation, (3) generating initial themes, (4) reviewing themes, (5) refining, defining, and naming themes, and (6) producing the report [28]. We also adapted our method according to Campbell and colleagues (2021) process with applied qualitative health research [29].

Focus groups were transcribed verbatim, and the first two steps of analysis were completed independently by the first, second, third, and fourth authors (T.-M.H.H., W.-J.H., B.A.L. and K.M.L.). T.-M.H.H. and B.A.L. both relied on critical intersectionality theory-driven approach to familiarize and generate codes while W.-J.H. and K.M.L. used a trauma-informed data-driven method. In other words, T.-M.H.H. and B.A.L. explored and interpreted the data based on assumptions that participants’ lived experiences provided unique meaning based on the intersections of their social positionings and systemic inequities they faced in society and healthcare, without generalizing across their racial or ethnic groups. Using a trauma-informed framework, W.-J.H. and K.M.L. relied on the assumption that all participants had traumatic experiences based on their minoritized identities. W.-J.H. and K.M.L. coded participant responses with attention to the cumulative and ongoing trauma experiences of participants in their healthcare interactions.

The first four authors attended weekly meetings to discuss their progress, review initial codes, and reflect on similarities and differences in an iterative process, which yielded approximately 24 concurrent initial codes; over 1000 new codes were generated across eight focus groups among these four authors. In the third phase, all five authors dedicated two meetings to sorting and summarizing semantic themes, which included three overarching themes within interpersonal interactions, seven themes within clinical and institutional challenges, and two themes about systemic and policy difficulties during COVID-19. Then, T.-M.H.H., W.-J.H., B.A.L. and K.M.L. each independently examined latent themes based on implied and shared meanings based on the list of 12 semantic themes that were consistent across eight focus groups. In the fourth and fifth phases, all authors continued to meet, review, refine, and name themes in an iterative manner, which resulted in three main latent global themes. All authors also engaged in a reflexive process throughout the data collection and analysis.

### 2.4. Reflexive Statements

Our research team comprises five cisgender people, of whom three are of Asian descent (Asian and Asian American), one of European descent (White), and one of African descent (Black/African American). The first and last authors both have three children and also gave birth during the COVID-19 pandemic. Our professional identities include two psychologists, two social workers, and one health communication expert. These social and professional identities informed our inclusion of intersectionality theory and trauma-informed frameworks within our data analytic approach due to our expertise through both lived and learned experiences. As a team and as individuals, we recognize that our lived experiences and positionings do impact our presence in the focus groups, our selection of probing prompts, and our interpretations of the results. Our findings are therefore interpreted in the context of public health, patient-researchers’ experiences, and our intersectional identities. 

## 3. Results

Our findings yielded three major latent qualitative themes that BIPOC women noted were common in their perinatal healthcare experiences during the COVID-19 pandemic: (1) an overwhelming lack of support from providers, (2) experiences of blame and shame, and (3) difficulties navigating institutional policies that were unclear or ever-changing during the COVID-19 pandemic. Three global themes, along with illustrative quotes relevant to each theme and the participants’ recommendations, are presented in Table 2.

### 3.1. An Unprecedented Lack of Support

The majority of participants felt an overwhelming lack of support in their perinatal period during COVID-19. The participants expressed several emotions, including but not limited to a lack of excitement, immense anxiety, uncertainty, fear, and a strong sense of isolation as a result of COVID-19 contact restriction policies implemented across healthcare clinics. These emotions were tied to a variety of factors, including their physical and social surroundings during the pandemic, less in-person support from partners/family, and less engagement with healthcare providers. For example, a participant expressed the emotions she grappled with during her perinatal experience:

*Definitely isolating, you know, no one coming to visit you, and then when you’re home, you know, no one’s coming to visit you then, only maybe immediate family, and that’s it. And even then, in all, it was very short.*

Overall, there was a general sentiment that being pregnant during a pandemic was a particularly isolating experience that included grief and loss. Given the contagious nature of COVID-19, many participants had little to no contact with loved ones throughout their pregnancy and post-delivery, causing them a great deal of anxiety from not having regular contact with their support system. Furthermore, the experience of feeling isolated was not unique to interpersonal relationships. Participants also recognized that the interactions with their healthcare providers were anything but the norm in preparing for the birth of their child. This issue might not be unique during the pandemic, but it was worsened because of COVID-19 policies as many participants expressed receiving minimum or limited support from their providers.

The findings revealed that perinatal BIPOC women experienced an overwhelming lack of support during their perinatal period during the pandemic, which worsened their feelings of isolation and enhanced powerlessness during pregnancy. For many of these BIPOC women, seeking perinatal care within a White-dominant healthcare institution was already a traumatic experience because they are not often treated the same as White patients, and the further lack of support they perceived during the COVID-19 pandemic could have exacerbated that traumatic experience.

### 3.2. Continued Blaming and Shaming

Similarly, participants expressed feeling judged by others regardless of the actions they chose to take during the perinatal period. The experience of blaming and shaming perinatal people was not new, but the pandemic worsened and allowed for new ways to blame and shame perinatal women. At the time when new vaccines were first made available, the general public was unable to access any data on pregnant people and young children. Subsequently, participants felt as though no matter their choices, such as whether they decided to receive the COVID-19 vaccination or not, they were harshly judged by others. For example, one participant shared:

*I felt like I might have been discriminated a little bit against, you know, because I chose not to, because I didn’t know what effects it would have on my child.*

Additionally, it was important to note that women’s pregnancy or delivery needs might no longer be the priority during the pandemic and that medical providers might not take the time to treat them with humanity and dignity, especially for BIPOC women. For example, with the COVID-19 testing protocol in place, there was an emphasis on getting every person tested in the midst of treatment, even when they were having a contraction. Thus, BIPOC women received messages and actions that communicated that COVID-19 testing trumped everything, including their pain, and they needed to wait for relief. This also highlighted the lack of consistent standards of care being implemented nationally during the pandemic given the conflicting COVID responses in the U.S. healthcare system. 

Based on the responses from participants, it appeared that the pandemic exacerbated existing stressors for BIPOC women and created new opportunities for blaming and shaming, which also heightened their sense of isolation and uncertainty. Our perinatal BIPOC women expressed the societal pressure that emphasized the mothers’ roles and responsibilities. This issue was not new given the patriarchal values embedded in our healthcare system, but during the pandemic, this added societal pressure also increased BIPOC women’s vulnerability to experiencing additional trauma on top of carrying a child. 

### 3.3. Institutional Policies Impacting Perinatal Experience

Overall, the participants were outspoken about how institutional policy changes in the healthcare system during the pandemic impacted the care they received and their overall perinatal experience. Furthermore, it was evident to these participants that the policies enacted by the healthcare system were done so without the input of the people affected by the healthcare system. Leading up to delivery, participants recognized the deep impact COVID-19 had on their perinatal experience, which included but was not limited to: a lack of access to treatment, proper support, resources, and education, the trauma of navigating the medical system by themselves, the restricted visitation policy at appointments, unnecessary appointments without rationale, and a lack of communication between providers at the same institution. For example, one participant expressed her concern:

*I don’t want to get exposed to COVID or anything, but on the other hand, it’s like there were no childbirth classes, and I was like, how do you not have childbirth classes? And so, I had to do research to find one.*

Due to the COVID-19 institutional policies, many participants described their perinatal experiences as inconvenient and out of their control. Many of the perinatal BIPOC women identified that the quality of care they received was compromised as a result of the institutional policies during COVID-19. Specifically, participants expressed their struggle with establishing regular perinatal treatment in the early stages of their pregnancies, lacking access to perinatal-related knowledge due to cancellations of informational workshops, and lacking access to proper telehealth support in the hospital. In addition to the quality of perinatal visits and appointments, some people further indicated that policies developed in response to COVID-19 impacted their ability to carry out their birthing plan. Although these outcomes were evidence of the fact that healthcare institutions did not have proper institutional policies in place to support perinatal people, the lack of clear guidelines from federal and national healthcare agencies compounded the systemic problems during the pandemic.

Overall, participants endorsed difficulties in navigating institutional policies during the COVID-19 pandemic; these policies deeply impacted their perinatal care experiences. The institutional policies made people’s pregnancies less about the celebration of birth and more about the management of trauma and loss.

### 3.4. Patient-Centered Recommendations from People with Lived Experience 

Toward the end of each focus group discussion, we asked participants what they would recommend to healthcare providers or the healthcare system more broadly that could have improved their perinatal experiences and made them feel safer. Participants identified two major areas for improvement: interpersonal interactions and institutional changes via training.

First, in terms of interpersonal interactions with healthcare professionals, participants indicated there was much to be done. Overall, the participants felt as though the healthcare providers they interacted with during their perinatal period could have made greater efforts in terms of providing social support, both emotional and informational. Specifically, participants expressed their need for emotional support and understanding from healthcare professionals when navigating a pregnancy during a pandemic. In a similar vein, participants felt as though because they were pregnant during a pandemic, they were receiving less information and guidance when it came to caring for themselves and preparing to care for their babies. This highlighted the need for centralized resources and support from healthcare systems, especially during a public health crisis. 

Related to the interpersonal recommendation, participants expressed that healthcare teams, ranging from the administrative staff checking in patients for their appointments to the clinicians delivering the babies, should be better equipped to provide compassion to patients. Specifically, participants recommended targeted empathy training for all healthcare providers.

## 4. Discussion

In this qualitative study, 41 participants described how COVID-19 exacerbated disparities in their access to care and overall prenatal care experience. The findings in this study align with numerous recent investigations on COVID-19 and alarming mental health issues and disparities in receiving care and support [30,31,32,33]. This study demonstrated the impact of structural barriers for BIPOC women who are racially minoritized perinatal people and highlighted systemic factors such as inconsistent information about COVID-19, lack of federal guidelines on standards of care, and increased discussions with providers about race and COVID-19. The experiences unveiled in this study identified contributors to structural disparities in the U.S. healthcare system and motivators for immediate change under the ongoing pandemic as well as future public health emergencies. 

Our study extended the intersectionality framework and trauma-informed perspective to understand the impact of systemic inequality on the experiences of perinatal BIPOC women. Similar to John et al.’s (2021) findings, we found consistent sentiments that healthcare professionals’ communication was lacking in information, patience, and empathy when participants tried to navigate pregnancy during a public health crisis [34]. Existing institutional issues worsened, such that appointments were fast or nonexistent, insurance or public aid accessibility was limited to specific services and providers, and communication was disjointed between different providers, even within the same hospital. These factors demonstrated difficulties in accessing healthcare services, resources, and support that are reflected in national trend data [30,31,32,33]; COVID-19 thus added additional barriers to seeking care. The COVID-19 pandemic has highlighted the trickle-down effect of institutional barriers and exacerbated stress for perinatal BIPOC women.

It was also clear that our findings aligned with Manca’s (2021) analysis of healthcare policies and responses to the COVID-19 pandemic, where the author stated that the voices and concerns of perinatal people were not part of the decision-making process. In fact, the COVID-19 pandemic responses added more burden and shame upon pregnant people while trying to minimize health risks and care for their babies without any support [1]. Even though these policies aim to reduce the universal risk of COVID-19 transmission, they disproportionally exacerbate disparities and at times put perinatal BIPOC people at more severe health risks and traumatic experiences due to their social positionalities. Our results add greater clarity to these findings by providing qualitative insight into the increase in mental health issues, depression, and anxiety symptoms among pregnant people during the COVID-19 pandemic [35]. After two years of the COVID-19 pandemic, nearly 1 in 4 pregnant people had experienced depression or anxiety. The cumulative impact of COVID-19 remains to be seen, but there is certainly a need to remain steadfast and proactively provide support for perinatal mental health.

In addition to feelings of isolation and lack of support, our racially minoritized perinatal women described their challenges in navigating multiple intersecting systems of oppression like racism, classism, and interpersonal gender violence. Our findings replicated what John et al. (2021) found in their sample of perinatal Women of Color from an urban Scottish health board area. One example of the interaction of systemic inequalities comprised gender inequality, reproductive justice, and vaccine bias [34]. Amidst inconsistent information about COVID-19 vaccines, our results showed the frequent shaming of pregnant people about whether they would get the vaccine, which was not equally distributed nor well-explained in terms of empirical data. This narrative also amplifies the societal power to strip away perinatal women’s right to bodily autonomy by enforcing women’s responsibility to minimize health risks for their babies. The tendency to blame perinatal women is not new, as we can see displayed in the current U.S. sociopolitical debate on women’s rights to abortion and contraception. These challenges continue to worsen perinatal health disparities and increase the collective intersectional trauma that perinatal BIPOC women face in navigating healthcare services.

To our knowledge, this is the first qualitative study that explored the lived experiences and challenges of perinatal BIPOC women during the COVID-19 pandemic in the U.S. grounded in an intersectionality and trauma-informed approach. The study, however, also has its limitations. Given that it was conducted online, only participants with access to devices with a video camera and audio recording functions and high-speed Internet could participate. Data also relied on personal narratives, so recall bias could be an issue when participants were asked to remember what their interactions were with the healthcare system and providers. In addition, the group effect could also apply, such that talkative participants’ narratives may have caused others to think similarly. This study also had a small sample size with smaller subsets of BIPOC women from different racial and ethnic backgrounds. Additionally, masking policies in healthcare settings may have also been a barrier to fully interpreting nonverbal communications in healthcare interactions, and we did not explore this aspect in the study. Lastly, we recognize that the moderators of the focus groups, the first and last authors, could be viewed as having more power than the participants, and thus their presence could have influenced how participants responded.

## 5. Implications

Perinatal BIPOC women face high risks of mortality and morbidity in the U.S. healthcare system, and this rate will continue to rise rapidly during the pandemic if our healthcare policies do not address systemic issues and harmful assumptions in serving this population. Our findings highlight the urgent need for change based on the input and lived experiences of these stakeholders. This study’s implications can be applied to addressing healthcare barriers and increasing access to healthcare in the event of future public health crises, such as emerging or re-emerging viruses and diseases (e.g., monkeypox, polio). 

We support the stakeholders’ recommendations to emphasize the need for systemic change in the role of providers because of their positions of power to create more positive impacts in serving BIPOC communities. The healthcare insurance system and access to healthcare services both must address the needs of perinatal BIPOC women who face multiple systems of marginalization which were worsened during a public health crisis. For example, in terms of access to care, healthcare coverage should extend from the prenatal to the postpartum period (up to one year after giving birth) to account for both personal and environmental risks associated with pregnancy and caretaking. As of now, there are only 23 states in the United States that allow a coverage extension under Medicare and Medicaid for the postpartum period [36]. Even more transformative, the burden of getting emergency care for pregnant people should be alleviated with less cost and administrative paperwork, especially during a public health crisis. Social administration, such as access to a Social Security office, resources, and local birthing support should be centralized and provided at local healthcare facilities, given limited contact and access during a pandemic. Responses to future public health crises also need to take into consideration existing structural barriers that BIPOC women already face when seeking healthcare services. Policies should be generated without compromising or worsening existing healthcare inequities. Training in trauma-informed approaches and cultural humility could be provided to increase transparency and trustworthiness, emotional support, understanding, and empathic healthcare interactions for both providers and patients. We note that trauma can happen to providers and staff as well as patients, as our medical system has been overloaded during the public health crisis. Research has documented elevated stress, trauma, and moral injury among healthcare workers during the pandemic [37] and to opportunity for supportive working environments to help reduce the distress. Thus, in the long term, creating a community of care with resources and support for all stakeholders within the healthcare system should be prioritized. 

## 6. Conclusions

As we emerge from the COVID-19 pandemic we are learning the profound impacts of structural barriers to obtaining full access to medical care and increased risk for mental health problems for BIPOC perinatal women. The impacts of COVID-19 will be long-lasting. However, the lessons learned from this public health emergency and stakeholders’ lived experiences can be immediately applied to create systems change. For instance, having a support person is a significant protective factor for perinatal women, especially for BIPOC perinatal women. Future studies can explore the role of support persons such as home visitors or doulas to assist in the perinatal period and reduce risks for perinatal mental health problems. Studies conducted in-person may also add additional insight.

## Figures and Tables

**Table 1:** Demographic Characteristics of the Participants (*N* = 41).

*Characteristic*	*N (%)*
**Gender**	
Woman	41 (100%)
**Age Range**	
18–24	10 (24%)
25–34	24 (59%)
35–44	6 (15%)
45+	1 (2%)
**Race/Ethnicity**	
Asian/Asian American Pacific Islander	8 (20%)
Black/African American	20 (49%)
Indigenous	1 (2%)
Latina	10 (24%)
Mixed Race/Multiracial	2 (5%)
**Geographic Location**	
Northeast	4 (10%)
Midwest	24 (58%)
South	5 (12%)
West	8 (20%)

**Table 2:** Organizing Themes and Basic Themes with Illustrative Quotes.

Organizing Theme	Basic Theme	Illustrative Quote Examples
**An Unprecedented Lack of Support**	**Lack of in-person support from partners/family**	*1. A, a Latina woman whose child is less than a year old, explained her social struggles during pregnancy:* “Definitely isolating, you know, no one coming to visit you, and then when you’re home, you know, no one’s coming to visit you then, only maybe immediate family, and that’s it. And even then, in all, it was very short, short lived. Like they were here, in my house, for like 20 minutes max, you know, just because we don’t want to risk anything, and you know, no baby shower, no, nothing like that either. So, it was definitely lonely.” *2. H, a Latina mother of one who was in her second trimester at the time of data collection, also revealed her isolating experience after delivery under the social distancing during COVID-19:* “How did it like affect, having COVID, how did COVID-19 affect the pregnancy or afterwards? I think it goes back to like the loneliness of it. I think again like for my experience, I had her right in the middle like when everything was still shut down. So, you know, nobody really met her right away. Nobody, you know, like my mom. I stayed at my mom’s because I had a C-section but, like, even when my mom grabbed her like, you know, we have nurses in the family. So, like she had on a gown and like, completely PPE on, just because she was so afraid to give it to her because not much was known about COVID of the time. So, I again, I just think very isolating, the whole thing with COVID was just very isolating.” *3. T, an Asian American woman with one child, described:* “I could just say I agree to about the good and bad of COVID like such as working from home. Some of the bad could be, you know, they really don’t want you going to the hospital. Like for example, my partner never even met my doctor. He did see one of the ultrasounds but that was with the genetic counselor. So, you know, I was all excited throughout the whole pregnancy in the beginning but you know, for a man not knowing and seeing it was kind of like, oh, it sucks. He can’t go to the doctor’s office with me to experience the ultrasound, so it took a little bit more time where he was able to go, which was another doctor so that, yeah, I agree with there’s good and bad and you know, cases are dropping but still the policies are that he’s not allowed in here.” *4. H, a Latina mother of one in her second trimester with her second child, shared a similar experience resulting from the restricted visiting policy at perinatal appointments during COVID-19:* “Like, no one can come to appointments with me and no one can go to ultrasounds with me still at the hospital that I am at. So I don’t have anything to base it off of, because it’s the same, like even though we’re in like the fourth or fifth wave.”
	**Lack of engagement with healthcare providers**	*1. Y, a Latina woman with three children, explained how even receiving the support she needed from healthcare providers was challenging:* “It’s the time of the pandemic, so we can’t just go out and talk to somebody. We’re all stuck at home, and we deal with it the way we can…I didn’t have any help from them [providers] whatsoever and the emotional support. I am currently pregnant, and they just told me that I’m expecting twins and, you know, they gave me a bunch of information that I wasn’t prepared for. They kept saying this information after another, that it was so overwhelming. And I’m like, I think I was just getting rid of that depression and now I’m gonna go back to it because of all this information. But I think I would love for the providers to be a little bit more sensitive. I know it’s hard for them to not to give all the information at once, but then also be mindful of taking that information to us and what can we do to cope with all this new information? And I don’t know. I just thought it was very overwhelming, I ended up with a headache instead of excitement. Don’t get me wrong but it was just so much that I didn’t even know how to digest that the new information.” *2. H, a Latina mother of one who was in her second trimester at the time of data collection, also revealed:* “I think right now, I think there just needs to be more like sympathy. I don’t think. And I don’t know if sympathy’s the right word, but more, maybe it is, like sympathy towards the situation. I mean, if you ask people who had kids five years ago, their experience is completely different than, you know, kids who were born during COVID. And I think that, that’s the one person that we have to talk to about all this. Like one, our fears of, what if we catch COVID? Or, like, just our fears of the different things that we have to do alone versus being, like, having our significant other with us. And, you know, I feel like it’s brushed off and it’s just kind of brushed off and just kind of pushed aside and it’s like, okay. Like, you know, you come in the appointment, do what you got to do, and get out. You know, and it’s like, well wait, all of these questions weren’t answered [by the providers], and I feel like I’ve been to three or four different ones now … and I think that there’s a lack of, um, lack of communication when it comes to that.” *3. R, a Black woman with one child, also shared:* “I ended up contracting COVID during my pregnancy, and just how they [the providers] handled that situation really pissed me off, for like their care, and I just didn’t really appreciate, like, how my concerns or worries were kind of just like, swept under the rug. So, I had to make some changes. I also decided to hire a doula after that experience, because I felt like me being a person of color, I mean, our experiences are not the same and they have a tendency to be traumatic. It’s already a traumatic experience, but they, they could be even more traumatic because we’re not often treated the same.”
**Blaming and Shaming of Perinatal People**	**Feelings of judgment from everyone regardless of the actions they chose to take during the pregnancy**	*1. T, an Asian American woman with one child, reflected on this very concern about the stigma around vaccination in retrospect after giving birth:* “I was actually really glad that the vaccine wasn’t available to me until after I gave birth because I feel like I probably wouldn’t have gotten it while pregnant, and I didn’t get vaccinated until several months after it was available to me because I was still breastfeeding and there wasn’t a lot of research on it and I just wasn’t sure. And for me, I’m like was working from home. I’m like, I’m not going out anywhere so I would rather reduce my risk that way and just not be vaccinated. But that judgment from other people, I felt that too.” *2. K, a Black woman with one child, described:* “But during the time, while I was delivering, the vaccine came up a lot and I’ll be honest, I was scared. You know, I didn’t know how the vaccine would harm or help or what reaction it would have to my baby. No, at the time they have the vaccine out for the adults but not for kids when I was delivering. So I was just scared, so I just like, “No, I don’t want it because I’m not sure what effects it would have on my child.” And I kind of got like looks you know, there’s a thing with unvax people versus vax people. And you know, the unvaxxed people are kind of looked at as “Oh, you’re the problem,” you know, but as a woman, you know, carrying a child, your first thoughts are always what’s best for my baby. You know, you’re always going to put your baby over you, you know, and I felt like I might have been discriminated a little bit against, you know, because I chose not to, because I didn’t know what affects about my child.”
	**Feeling blamed for prioritizing own needs**	*1. K, a Black woman with two children, shared her experience:* “I just wanted to add that in the moment of apologizing, you were also trying to bring a baby into this world. I was getting [an] IV in my left arm and the COVID test the exact same time. I was having contraction, and everyone was working. I was still a human and I was like “I’m in pain, can you stop?” and they didn’t listen and it was so bizarre. Everything happened, you know, the outcome was great. But in that moment of five minutes of turmoil, I was afraid that like, I was going to do something wrong or like do the wrong thing and then they [the providers] would like, just drop the ball. That did not happen, that was just my fear because Black women do die at an exponential rate than other.”
**Institutional Policies Impacting Perinatal Experience**	**Lack of access to proper treatment, support, resources, and education**	*1. N, a multiracial AfroLatina woman and mother of one child, shared her experience as a result of the institutional policies in place for COVID-19:* “When I had my 20 weeks check-up, they said, “oh, you could FaceTime your significant other or you can FaceTime a family member,” but then their computer went down. So it was always like ‘This is news that I want to share,’ but I’m really alone. Like I can’t share this. I can’t let somebody hear the heartbeat right now, it was a very isolating experience.” *2. M, an Indigenous woman with one child, revealed her struggle to establish regular perinatal treatment in the early stage of her pregnancy:* “I was losing 15 pounds a week… and they [the ER providers] kept telling me that what I was going through is normal, and it clearly was not normal, and it was just really frustrating because I have to go to the emergency room maybe six times before they would get me in to schedule an appointment.” *3. J, a Black woman in her third trimester at the time of data collection, also expressed her concern:* “I don’t want to get exposed to COVID or anything, but on the other hand, it’s like there were no childbirth classes and I was like, how do you not have childbirth classes? And so, I had to do research to find one.” *4. K, a Black woman with one child, described how she had to make some difficult decisions and strayed from her desired plan:* “So, I ended up having to have an emergency C-section, going away from my plan and with COVID policies in place with the hospital. My entire birthing team was not able to come. So, I had to under emergency circumstances, like try to make the best decision of who would go with me into the hospital.”
	**Lack of flexibility for treatment and other administration services**	*1. M, an Indigenous woman, described:* “I just felt like some of them were a little unnecessary and you know, if I don’t have to go into a doctor’s office during COVID, why make me, you know, I kind of felt obligated to go and they were like you need to go get checked just to make sure everything’s right, and you’re not having other issues and rule this out rule that out, but if that’s not the case for me, I don’t need to go, you know, what’s the point?” *2. N, a multiracial person, described:* “He’s [the baby] Asian, Latino and Black so I want to give my child an ethnic name so that he can relate to culture, so I told them [the hospital staff] to add two accent marks.… They’re like, ‘Okay well if you put the accent marks in, then you’re not going to get a Social Security number from us. We’re not even going to register for that. You’re going to have to do that on your own.’”
**Recommendations**		*1. H, a Latina woman with one child who was pregnant at the time of data collection, discussed how greater emotional support and understanding is needed from healthcare professionals when a patient is navigating pregnancy during a pandemic:* “I think right now, I think there just needs to be more like sympathy...I mean, if you ask people who had kids five years ago, their experience is completely different than, you know, kids who were born during COVID. And I think that, that’s the one person that we have to talk to about all this. Like one, our fears of, ‘What if we catch COVID?’ Or, like, just our fears of the different things that we have to do alone versus being, like, having our significant other with us. And, you know, I feel like it’s definitely brushed off.” *2. N, a multiracial AfroLatina woman with one child, recommended that healthcare providers take the time to solicit information for mothers:* “One thing that I would recommend for physicians to do is to provide information before it’s asked. Ask the person like, ‘Oh, so are you looking into finding out the gender? Do you know what kind of diet you should be taking? Do you want to breastfeed? This is when your milk will come in,’ so that you’re not in the moment and you’re panicking like, ‘Oh, I can’t do this. I can’t do that,’ because we can if we are informed.” *3. K, a Black woman with one child, recommended that empathy training could be provided for all those who interface with patients:* “I think one good solution would be to have maybe a training.... When you’re going into the healthcare field, no matter what your title is because I do feel like we’re looked at more so as dollar sign than actually humans, you know, so I understand that life isn’t great. We had a bad day or, you know, your patient overload is ridiculous, and we’ll call it off and now you have double the patients, you know, things happen, life happens, but still we’re all here, you know, to get it care. We would like to just treat it with a little more compassion. So I think that would be a training on how to just be more, regardless of the circumstances, be more sympathetic and compassionate to your clients. That might be a step in the right direction...maybe just a class on treat your clients as if they’re your children, you know, [as] if they’re your family members, you know, just be kind and you know considerate to them.”

## Data Availability

The data presented in this study are available on request from the corresponding author. The data are not publicly available due to privacy.
